# Changes in human health parameters associated with an immersive exhibit experience at a zoological institution

**DOI:** 10.1371/journal.pone.0231383

**Published:** 2020-04-17

**Authors:** Audrey A. Coolman, Amy Niedbalski, David M. Powell, Corinne P. Kozlowski, Ashley D. Franklin, Sharon L. Deem

**Affiliations:** 1 Institute for Conservation Medicine, Saint Louis Zoo, St. Louis, Missouri, United States of America; 2 Brown School, Washington University in St. Louis, St. Louis, Missouri, United States of America; 3 Conservation Audience Research and Evaluation, Saint Louis Zoo, St. Louis, Missouri, United States of America; 4 Department of Reproductive and Behavioral Sciences, Saint Louis Zoo, St. Louis, Missouri, United States of America; 5 Association of Zoos and Aquariums Reproductive Management Center, Saint Louis Zoo, St. Louis, Missouri, United States of America; Iwate Medical University, JAPAN

## Abstract

Zoological institutions often use immersive, naturalistic exhibits to create an inclusive atmosphere that is inviting for visitors while providing for the welfare of animals in their collections. In this study, we investigated physiological changes in salivary cortisol and blood pressure, as well as psychological changes among visitors before and after a walk through the River’s Edge, an immersive, naturalistic exhibit at the Saint Louis Zoo. Study participants had a significant reduction in salivary cortisol and blood pressure after walking through the exhibit. Psychological assessments of mood found that most visitors felt happier, more energized, and less tense after the visit. Additionally, participants who spent more time in River’s Edge, had visited River’s Edge prior to the study, and had seen more exhibits at the Zoo prior to entering River’s Edge experienced greater psychological and/or physiological benefits. We conclude that immersive, naturalistic exhibits in zoos can elicit positive changes in physiological and psychological measures of health and well-being and argue for a greater scientific focus on the role of zoos and other green spaces in human health.

## Introduction

Stress and illness have a complicated relationship, yet there is strong evidence that acute and chronic stress may lead to long-term negative outcomes on an individual’s overall health and quality of life [1, 2, 3, 4]. Hans Selye first defined the term stress in 1936 as “the non-specific response of the body to any demand for change” [5]. Any disruption to homeostasis can be considered stress; yet individuals may perceive the same stimuli differently [6, 7]. These non-specific responses may manifest as changes that are psychological, physiological, or both, as interactions occur across the mind–body continuum and can be either health promoting or health damaging [1, 8]. The most commonly used biological indicators of stress in humans have traditionally been elevated blood pressure (hypertension) and increased cortisol levels [8].

One predictor of general health and level of stress is access to green spaces [9, 10, 11]. Studies have supported that connection to nature may relieve stress in humans [11, 12, 13, 14]. Contact with nature has been shown to provide measurable psychological and physiological health benefits that are associated with relaxation and restoration from stress, yet many people living in an urbanized setting lack access to green spaces [12, 14, 15, 16, 17]. An individual’s exposure to nature and the resulting human health benefits may provide positive public health outcomes [9, 10, 11]. The existence of human health benefits derived from experience in nature is one facet of the growing understanding of how environmental, human and animal health are inter-connected within a framework of One Health [18, 19, 20, 21].

One Health is an initiative based on the understanding that the health of all life is inextricably linked and involves multi-disciplinary collaboration to optimize the health of people, animals, and the environment [19, 20, 22]. In the last decade, many Association of Zoos and Aquariums (AZA) institutions have been participants in this initiative [21, 23, 24]. For instance, numerous zoos focus on the conservation of animal species and wildlife habitats, both important for ecosystem health and resilience, through their efforts both with collection animals as well as *in situ* with animals throughout the world [21, 23, 25, 26]. Additionally, naturalistic exhibits of animals and well-maintained natural green spaces on zoo campuses may provide health benefits for zoo visitors as has been demonstrated previously [27, 28].

Only a handful of studies have explored how zoos and aquariums provide direct benefits to human health. A study conducted at a Japanese zoo found that visitors experienced a decrease in blood pressure, walked over 6,000 steps, and reported an increased self-perceived quality of life during their visit [28]. Another study evaluated visitor stress during a touch tank experience with stingrays and found a decrease in psychological stress for participants [27]. A third study found reductions in heart rate, increases in self-reported mood, and higher interest in marine life among aquarium visitors, especially for individuals typically without access to natural environments [29]. The extent of these changes increased with increasing exposure to more animals [29].

In this study, we examined changes in health parameters among visitors associated with a walk-through of an immersive, naturalistic exhibit at a zoo by using physiological and psychological assessments. We predicted that there are health benefits that zoos provide to their visitors and that our study will help to identify ways in which zoos improve the health of their visitors. We measured blood pressure and salivary cortisol to assess changes in physiological stress and used a psychological tool to assess mood. Blood pressure measurement is a standard indicator of stress [30]. Changes in cortisol levels reflect the body’s adaptive response to challenging stimuli [31, 32]. Cortisol may increase regardless of whether the subject perceives the stimulus as positive or negative [33, 34, 35]. Decreases in cortisol are generally interpreted as reflecting the body’s return to homeostasis and the reduced perception of challenge [36]. Research on hormonal responses to acute changes in stress and affective state have been limited. However, a handful of studies have detected changes in cortisol production in response to short-term positive experiences. For example, salivary cortisol concentrations have been shown to decrease following participation in Hatha yoga classes, as well as sessions of relaxation exercises and music therapy [37, 38, 39]. Salivary cortisol analyses have been used previously in human studies to understand stress levels in relationship to exposure to green spaces with evidence showing that spending short amounts of time in forested areas can decrease cortisol production in comparison to urban areas [11, 40].

We used the University of Wales Institute of Science and Technology Mood Adjective Checklist (UMACL) to assess psychological parameters of zoo visitors’ mood [27, 41]. This tool includes three dimensions: hedonic tone (HT: happy to sad), energetic arousal (EA: energized to tired), and tense arousal (TA: tense to relaxed), which have been validated as being sensitive to a generalized stress response [41]. We predicted that blood pressure, salivary cortisol, and indicators of negative arousal (i.e., TA) would decrease while indicators of positive arousal (i.e., HT & EA) would increase as a result of walking through an immersive, naturalistic exhibit at a zoo.

## Materials and methods

### Experimental procedure

We conducted the study from April to June 2018 in the River’s Edge exhibit at the Saint Louis Zoo (St. Louis, MO). River’s Edge is a 1.21 km trail on which visitors walk from entrance to exit along a heavily planted, winding trail featuring naturalistic habitats. Animals housed along the trail include: Andean bear, (*Tremarctos ornatus*), bush dogs (S*peothos venaticus)*, capybaras (*Hydrochoerus hydrochaeris)*, black rhinoceros (*Diceros bicornis)*, painted dogs (*Lycaon pictus)*, red river hogs (*Potamochoerus porcus)*, bat-eared fox (*Otocyon megalotis)*, hippopotamus (*Hippopotamus amphibious)*, spotted hyenas (*Crocuta crocuta)*, cheetahs (*Acinonyx jubatus)*, dwarf mongoose (*Helogale parvula)*, Asian elephants (*Elephas maximas*), Malayan sun bear (*Helarctos malayanus*), and a variety of North American fish species.

We used a systematic random sampling technique to recruit participants from Zoo visitors as they entered River’s Edge between 11:00 AM and 2:00 PM Monday through Friday. We selected this time of day to avoid the morning peak in cortisol production [42, 43]. A researcher stationed at the exhibit entrance approached every second adult (alternating assumed genders), explained the basic study protocol, and invited their participation. We sampled an equal number of men and women. Based on previous data, participants were also required to meet the following criteria: non-nicotine users, be between the ages of 18 and 55 years, had not consumed caffeine, food or gum in the previous hour, or alcohol in the previous 12 hours [44, 45]. Adults who appeared to be chaperoning a school group or field trip were not included in the random sampling. After all inclusion criteria were met and an individual agreed to participate, the person was escorted to a table nearby and introduced to a second researcher. An additional Zoo staff member or volunteer stood nearby to educate and/or entertain any additional members of the individual’s party during the period of data collection.

### Data collection

The second researcher explained the study protocol, consent form, and purpose of the study. After giving informed consent, each participant completed a pre-visit questionnaire form. This questionnaire included the following: first visit to River’s Edge (Yes or No), age, parking experience rating (scale from 1 to 10, where 1 is “poor” and 10 is “excellent”), number of exhibits seen prior to River’s Edge, and time of arrival at the Zoo. Each participant then answered the pre-UMACL, which included 24 mood adjectives, with eight corresponding adjectives to each of the three dimensions (see statistical analyses).

After completing all forms, we then collected physiological data. Prior to saliva collection, each participant rinsed their mouth with water (supplied by the researcher) and placed a SalivaBio Oral Swab (Item # 5001.02, Salimetrics, State College, PA) under their tongue for two minutes (as specified by the manufacturer), while sitting quietly. After two minutes, the swab was inserted into a Saliva Storage Tube (Item # 5001.05, Salimetrics, State College, PA), either directly by the participant or by the researcher without direct contact. After collection, saliva samples were placed in a cooler on ice packs for no longer than two hours, and then brought to the lab for processing.

Next, a trained researcher took the participant’s blood pressure using an OMRON M6 AC (HEM-7322-E) upper arm blood pressure monitor in accordance with the IRB-approved protocol [46]. The American Heart Association recommends a five-minute rest period prior to blood pressure collection to normalize pressure [47, 48]. By having participants fill out forms and complete cortisol collection prior to taking their blood pressure, we ensured that participants fulfilled their five-minute rest period. Additionally, we asked participants to sit quietly without their cell phones for two minutes after cortisol collection, averaging a total of seven minutes resting time. Per manufacturer directions, blood pressure was recorded using the participant’s left arm placed flat on a table with both feet flat on the ground. Prior to the study, all researchers were given formal training by Zoo medics on using the OMRON M6 AC (HEM-7322-E).

Finally, we asked participants to wear a colorful lanyard to identify themselves to the researchers at the end of the exhibit trail. Participants were also instructed not to consume any food or drinks (other than the boxed water provided) and refrain from chewing gum during their walk through the exhibit. We invited participants to enjoy their time in River’s Edge at their normal pace without any time restrictions.

Once the participant finished walking through River’s Edge, they performed a similar suite of tests, including measurement of blood pressure and collection of saliva samples, and a post-visit questionnaire. This had the following questions: gender (as identified by the participant), party size, number of kids in party under 14 years old, incidence of having a staff interaction, animal that they found most engaging (if any), whether they had a close up encounter with that animal, and looked into the animal’s eyes. Each participant then answered the post-UMACL. A researcher timed and recorded each step in the data collection process. The total number of minutes spent in River’s Edge was calculated, total number of minutes spent in the Zoo prior to entering River’s Edge and outside air temperature during River’s Edge was recorded for each participant. Daily Zoo attendance was included as an additional variable.

Upon completion of the UMACL form, post-questionnaire survey, saliva collection, and blood pressure reading, participants received a $10 Zoo gift certificate.

This study was approved by Heartland Institutional Review Board (IRB) and all principal investigators completed the Collaborative Institutional Training Initiative (CITI) IRB training. The study was deemed expedited with written consent.

### Laboratory diagnostics

Saliva swabs within the storage tubes were spun in a centrifuge at 1500 g for 15 minutes to extract saliva from the swabs. Saliva samples were then pipetted into cryovials and stored at -20°C until assay. Salivary cortisol concentrations were measured using a commercially available enzyme immunoassay designed for human saliva (Item # 1–3002, Salimetrics, State College, PA). The assay has been validated previously, with a mean recovery percentage of 104.9 ± 2.0% (n = 8), and a mean dilution recovery percentage of 105.3 ± 1.4% (n = 4) (data provided by the manufacturer). The lower detection limit was 0.012 μg/dL and the upper detection limit was 3.0 μg/dL. Samples were assayed neat; any sample that was above the detection limits was diluted 1:10 with assay buffer and reanalyzed. All samples were assayed in duplicate. Mean intra-assay variation of duplicate samples was 8.0%; mean inter-assay coefficient of variation for two quality control pools was 6.1%.

### Statistical analyses

Descriptive statistical analyses were completed using IBM SPSS software v23 (IBM, Armonk, New York, USA). Descriptive statistics were used to determine if there was adequate variability in each variable for inclusion in the model described below. A paired sample *t*-test was used to calculate differences in means from pre to post for the six experimental measures, including systolic and diastolic blood pressure, cortisol, EA, HT, and TA. A Pearson’s chi-squared test was run on systolic and diastolic blood pressure categories (normal, elevated and hypertensive) to test for differences in proportions from pre to post.

Regarding the UMACL, participants marked how each adjective reflected his or her current mood using a four-point scale (1 = “definitely not,” 2 = “slightly not,” 3 = “slightly,” 4 = “definitely”). Within each set of eight adjectives, four represented the positive end of each dimension and four the negative end (Table 1). Responses for adjectives negatively related to each dimension were reversed, and sub scores for each dimension were obtained by summing the responses for each adjective [27, 41]. The same version of the UMACL was used pre- and post-River’s Edge experience.

**Table 1:** University of Wales Institute of science and technology mood adjective checklist (UMACL) for assessment of the psychological parameters of zoo visitors’ moods.

Item	Hedonic Tone (HT)	Energetic Arousal (EA)	Tense Arousal (TA)
Happy	+		
Dissatisfied	-		
Energetic		+	
Relaxed			-
Alert		+	
Nervous			+
Passive		-	
Cheerful	+		
Tense			+
Jittery			+
Sluggish		-	
Sorry	-		
Composed			-
Depressed	-		
Restful			-
Vigorous		+	
Anxious			+
Satisfied	+		
Unenterprising		-	
Sad	-		
Calm			-
Active		+	
Contented	+		
Tired		-	

“+” and”-”indicate whether the adjective contributed to the positive or negative end of the mood dimension, respectively.

Inferential statistical analyses were performed in SAS® Studio 3.7 (SAS Institute Inc., Cary, NC, USA). Prior to analysis, study participants were assigned to two blocks based on the time of day they entered River’s Edge (prior to 12:30 PM vs. after 12:30 PM), controlling for effect of time of day on the response variables measured. The effects of possibly associated factors on systolic and diastolic blood pressure, salivary cortisol concentrations, TA, EA, and HT were all evaluated using a generalized linear mixed model within the MIXED procedure. Fixed categorical factors included time (pre or post River’s Edge experience), gender (male or female), first visit to River’s Edge (yes or no), found the elephants the most engaging (yes or no), had a staff interaction (yes or no), had a close up encounter with the animal found to be most engaging (yes or no), and looked into this animal’s eyes (yes or no). Quantitative variables included air temperature, age, daily Zoo attendance, party size, number of kids in party under 14 years old, parking experience score, total number of exhibits seen prior to River’s Edge, total number of minutes spent at the Zoo prior to River’s Edge, and total number of minutes spent in River’s Edge. These variables were selected based on sufficient variation in subject responses and previous data that support their impacts on visitor experiences. For example, at the Saint Louis Zoo, visitors report parking as the most experienced inconvenience during a Zoo visit [49]. Additionally, it was demonstrated in a previous study that the extent of eye contact with animals at a zoo may significantly impact a visitor’s emotional response [50]. Lastly, it was shown that elephant viewing may have an affective response in visitors [51].

Individual ID and block (described above) were included as random factors, as well as the interaction terms between time, and the fixed and quantitative factors. Non-significant interaction terms between time and quantitative factors were removed to generate a single slope term for the final models. All other variables, regardless of statistical significance, were included in the final models. Though the design of the study is repeated measures in nature, there were only two measurements made within each subject, therefore a simple variance component (VC) covariance structure was used. The Tukey method for multiple mean comparisons was used to adjust *p* values when simple effect means were compared. Differences were declared significantly different using an alpha = 0.05. The block effect was not significant in any of the analyses and therefore not explored further within this study. All data met the assumptions for parametric tests based on visual examination of the residuals.

## Results

Over the eight week period of data collection, 1,656 Zoo visitors were approached and invited to complete the study. Of these, 1,052 chose not to participate and 402 agreed to participate, but did not meet all of the qualifications and were not included. There were 202 participants who completed the study for an overall response rate of 12%. We excluded data from 16 individuals, leaving 186 participants for the final analysis. Reasons for exclusion included not finishing the post-study questionnaire, drinking something other than water during their River’s Edge visit, too little saliva collected either during between pre or post swab, or not fully completing the mood adjective checklist in either the pre or post study survey. Demographic and zoo experience-related descriptive statistics are provided in Table 2. Our sample was nearly balanced with regard to gender and ranged in age from 18 to 55 years. Party size averaged 3.1 individuals but ranged as high as 25 individuals. On average, parties included one child under the age of 14. Most visitors reported a positive parking experience prior to entering the Zoo. Most commonly, visitors had been at the Zoo for over an hour and had visited four other exhibits at the Zoo prior to entering River’s Edge. Visitors spent approximately a half hour in River’s Edge (range 9–141 minutes) and saw an average of nine species (range 2–19). Most participants had been to River’s Edge previously and were nearly evenly split on whether or not they found the elephants to be the most engaging animal. Participants reported that they were able to have an up-close experience with the animal they found most engaging, but only about a third of participants reported looking into that animal’s eyes. Most visitors did not have an interaction with a staff member during their visit to River’s Edge.

**Table 2:** Demographic and zoo experience-related descriptive statistics for the health study participants of an immersive exhibit experience at a zoological institution.

Variable	Mean ± *SD*	Min	Max
Participant age (in years)	28.4 ± 8.4	18	55
Number of persons in party (including participant)	3.1 ± 2.9	1	25
Number of persons in party under age 14	0.7 ± 1.7	0	15
Parking experience rating (scale from 1 to 10, where 1 is “poor” and 10 is “excellent”)	8.0 ± 2.1	1	10
Total number of minutes spent at the Zoo prior to arriving at the River’s Edge	75.6 ± 61.9	1	267
Total number of exhibits seen prior to arriving at the River’s Edge	4.0 ± 3.7	0	13
Total number of animal species seen in the River’s Edge	8.7 ± 2.5	2	19
Total number of minutes spent in River’s Edge	31.5 ± 14.4	9	141
Outside air temperature (°F)	74.6 ± 10.1	39	93
Daily Zoo attendance	10,883 ± 3,251	3,313	19,116
	**Count (% of participants)**		
Gender:			
*Female*	91 (48.9%)		
*Male*	95 (51.1%)		
First ever visit to River’s Edge:			
*Yes*	62 (33.3%)		
*No*	124 (66.7%)		
Found the elephants the most engaging animal:			
*Yes*	96 (51.6%)		
*No*	90 (48.4%)		
Interacted with a staff member:			
*Yes*	30 (16.1%)		
*No*	156 (83.9%)		
Experienced the “most engaging” animal in its habitat close up:			
*Yes*	142 (76.3%)		
*No*	44 (23.7%)		
Looked into this animal’s eyes:			
*Yes*	63 (33.9%)		
*No*	123 (66.1%)		

### Predictors of blood pressure dynamics

**Fig 1:**
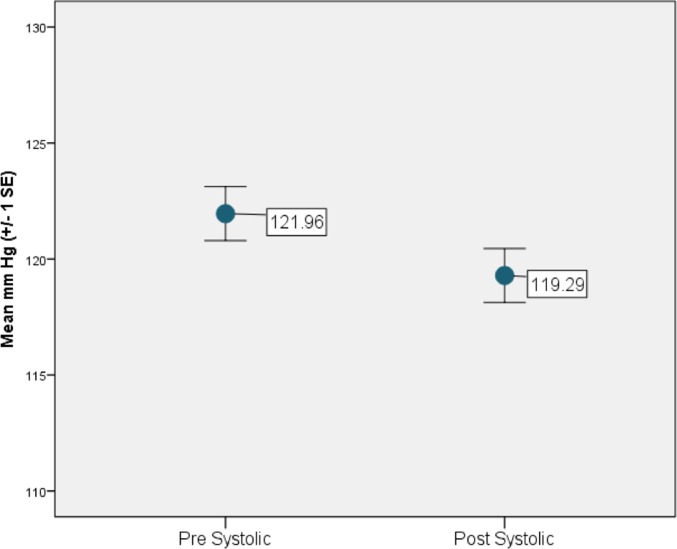
Study participant average systolic blood pressure before and after the River’s Edge experience at the Saint Louis Zoo.

**Fig 2:**
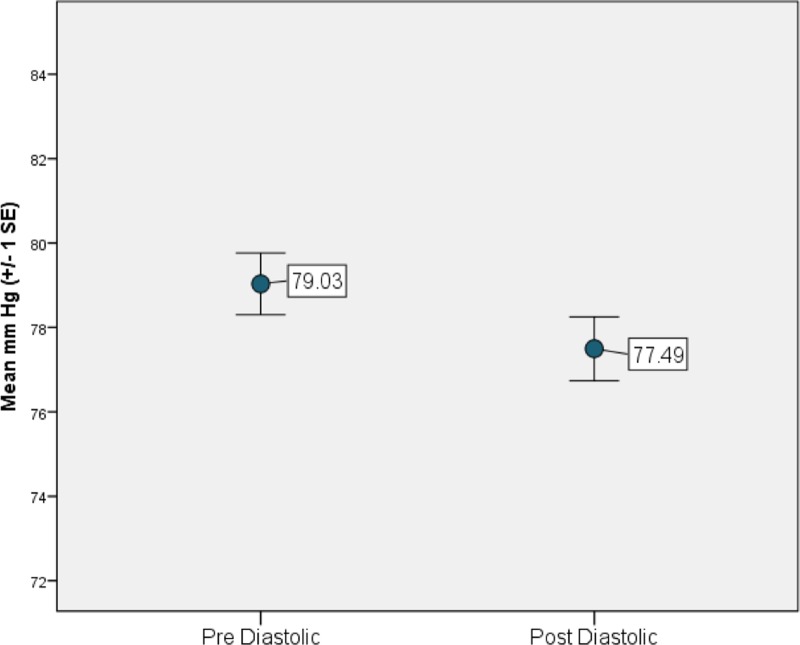
Study participant average diastolic blood pressure before and after the River’s Edge experience at the Saint Louis Zoo.

On average, both systolic and diastolic blood pressure significantly decreased from pre- to post- (over time) River’s Edge experience (systolic: *t*_185_ = 3.61, *p* = 0.0004, Fig 1 and diastolic: *t*_185_ = 3.07, *p* = 0.0024, Fig 2). This relationship was still significant when the data were analyzed as a generalized mixed model for systolic blood pressure (F_1,179_ = 4.51, *p* = 0.035) and marginally significant for diastolic blood pressure (F_1,179_ = 3.21, *p* = 0.075). Males had significantly higher systolic blood pressure and marginally higher diastolic blood pressure compared to females (F_1,179_ = 60.35, *p* < 0.0001 and F_1,179_ = 3.65, *p* = 0.058, respectively). Age was significantly associated with both systolic and diastolic blood pressure: higher blood pressure was associated with increasing age (r = 0.4565, t_179_ = 3.80, *p* = 0.0002 and r = 0.2763, t_179_ = 3.16, *p* = 0.0018, for systolic and diastolic blood pressures, respectively). Diastolic blood pressure was also affected by air temperature and party size. Lower diastolic values were associated with higher air temperatures (r = -0.1625, t_179_ = -2.30, *p* = 0.023) and larger party sizes (r = -0.5991, t_179_ = -2.14", *p* = 0.034).

**Fig 3:**
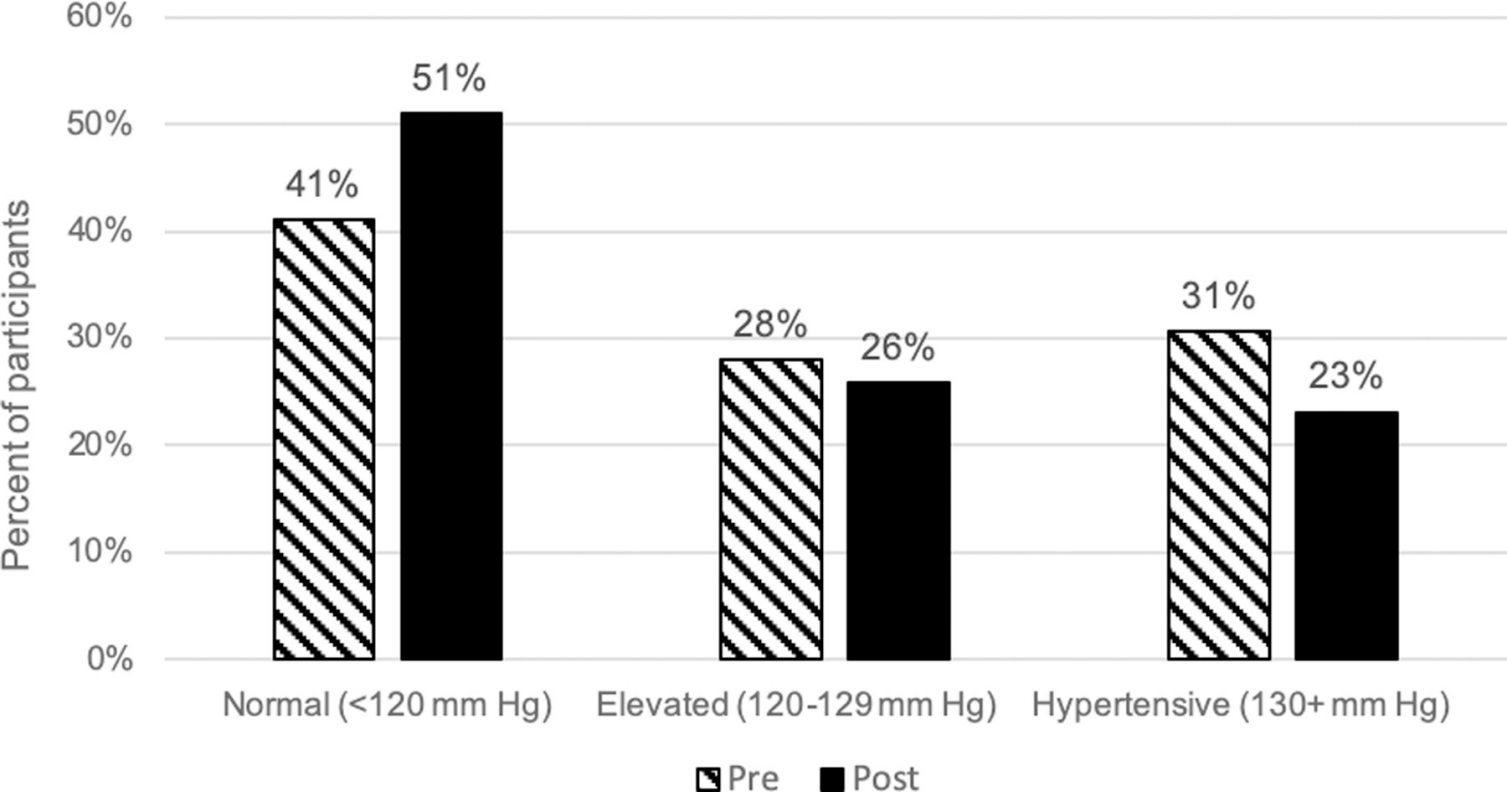
Percent of study participants categorized by systolic blood pressure categories, pre and post the River’s Edge experience.

**Fig 4:**
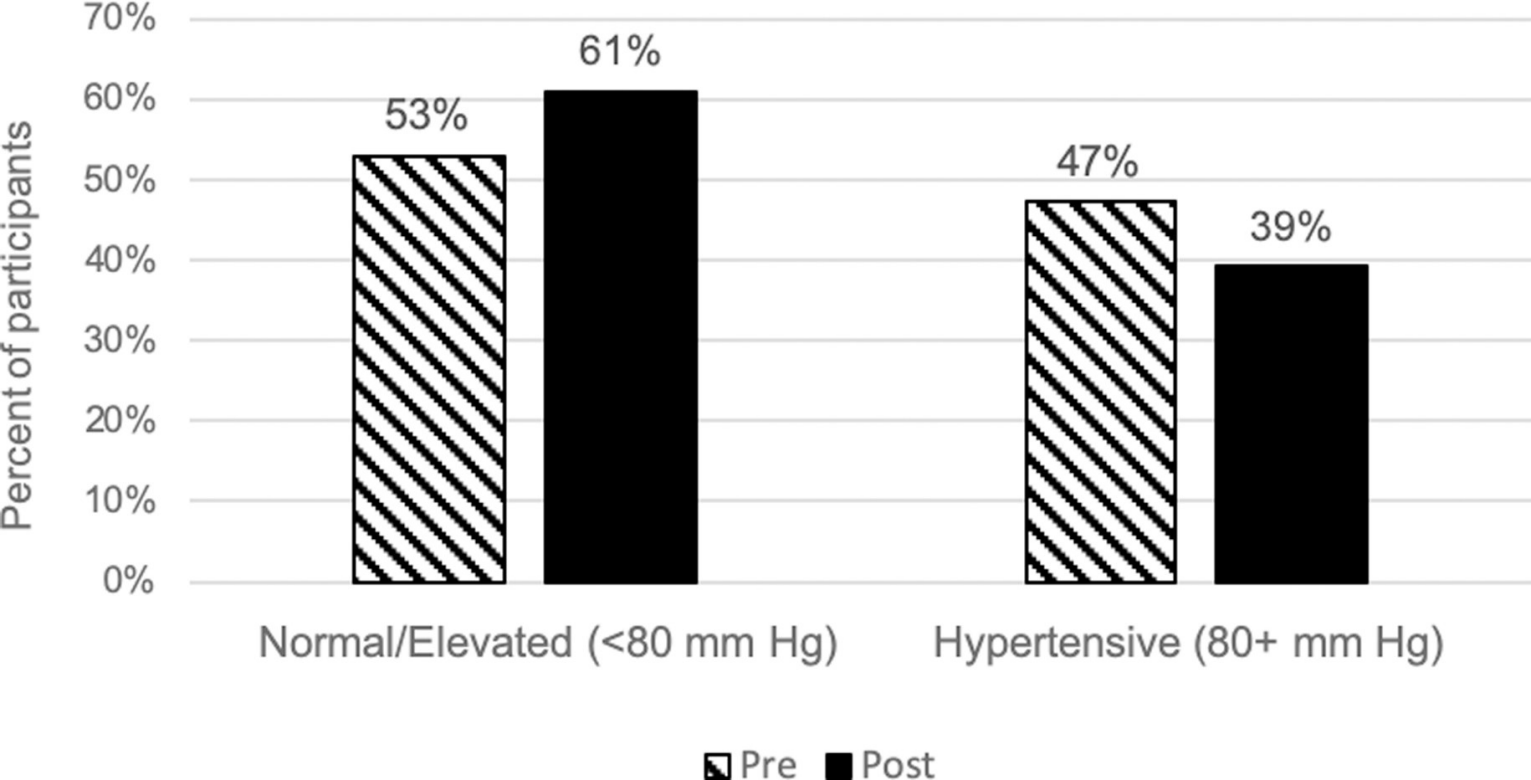
Percent of study participants categorized by diastolic blood pressure categories, pre and post River’s Edge experience.

During their River’s Edge visit, 23% of the 186 participants shifted from a higher systolic blood pressure category to a lower systolic blood pressure category with 17% of the total participants shifting down to the normal category (*x*^*2*^ = 4.004, df = 2, *p* = 0.135). Additionally, 15% of participants shifted from a higher diastolic blood pressure category to the normal diastolic blood pressure category (*x*^*2*^ = 2.464, df = 1, *p* = 0.116). We defined categories based on the guidelines of the American Heart Association, Inc. [52]. There was an increase in the total proportion of participants in the normal systolic blood pressure category post-River’s Edge experience (51%) compared to pre-River’s Edge experience (41%) (Fig 3). Additionally, there was a decrease in the proportion of participants in the normal diastolic blood pressure category post-River’s Edge experience (61%) when compared to pre-River’s Edge experience (53%) (Fig 4). However, the changes in proportions were found to be non-significant.

### Predictors of cortisol dynamics

**Fig 5:**
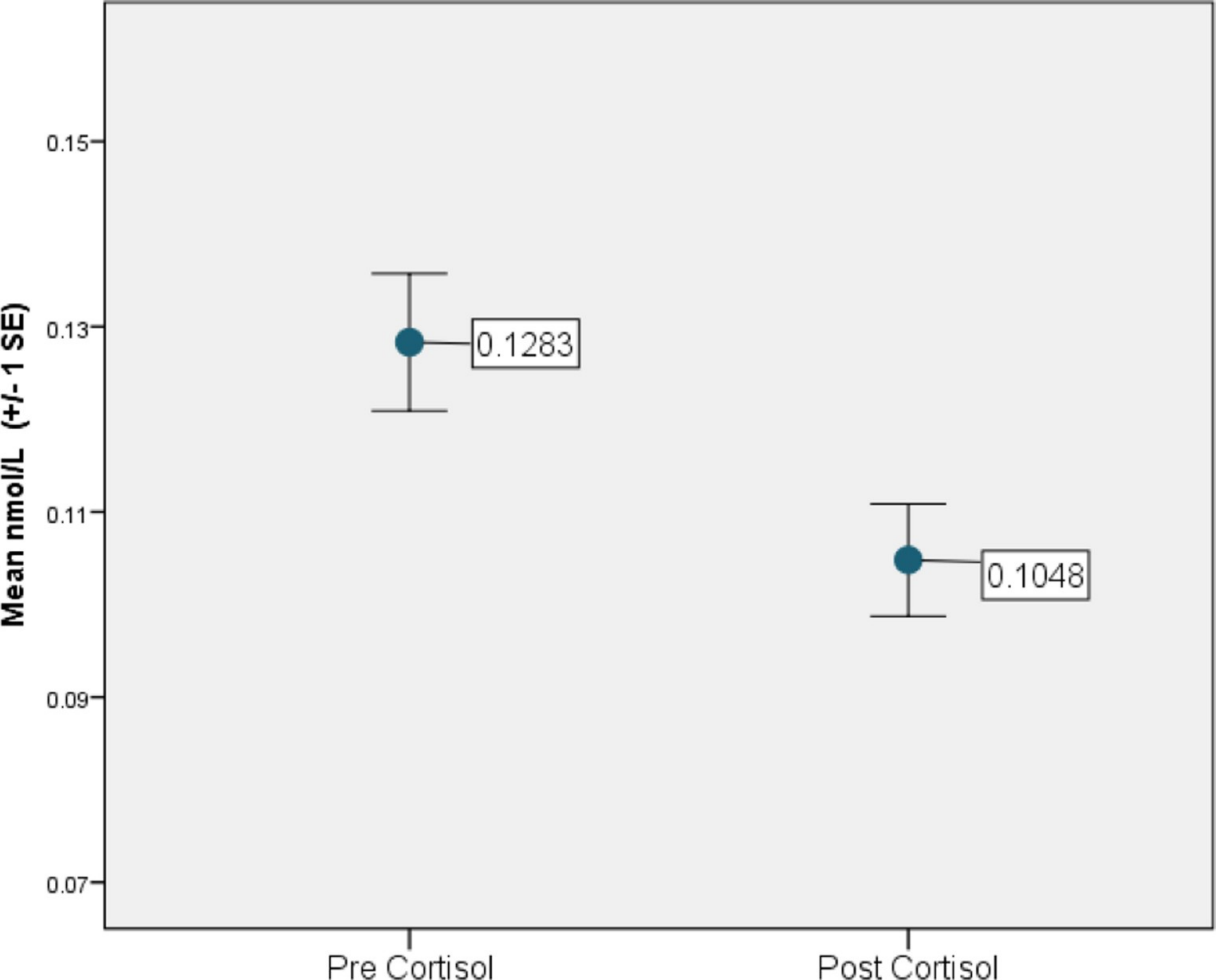
Study participant average salivary cortisol concentrations before and after the River’s Edge experience at the Saint Louis Zoo.

On average, salivary cortisol significantly decreased from pre- to post-River’s Edge experience (*t*_185_ = 3.76, *p* = 0.0002, Fig 5). This relationship was still significant when the data were analyzed as a generalized mixed model (F_1,178_ = 13.74, *p* = 0.0003). Participants who had visited more exhibits prior to River’s Edge had lower salivary cortisol upon arrival to the River’s Edge (r = -0.008, t_178_ = -2.61, *p* = 0.010). This association was no longer significant upon completion of the River’s Edge experience (r = -0.003, t_178_ = -0.99, *p* = 0.323). Although only marginally significant, the greater the amount of time a person spent walking through River’s Edge, the greater the observed decrease in cortisol (F_1,178_ = 2.92, *p* = 0.089).

### Predictors of hedonic tone

**Fig 6:**
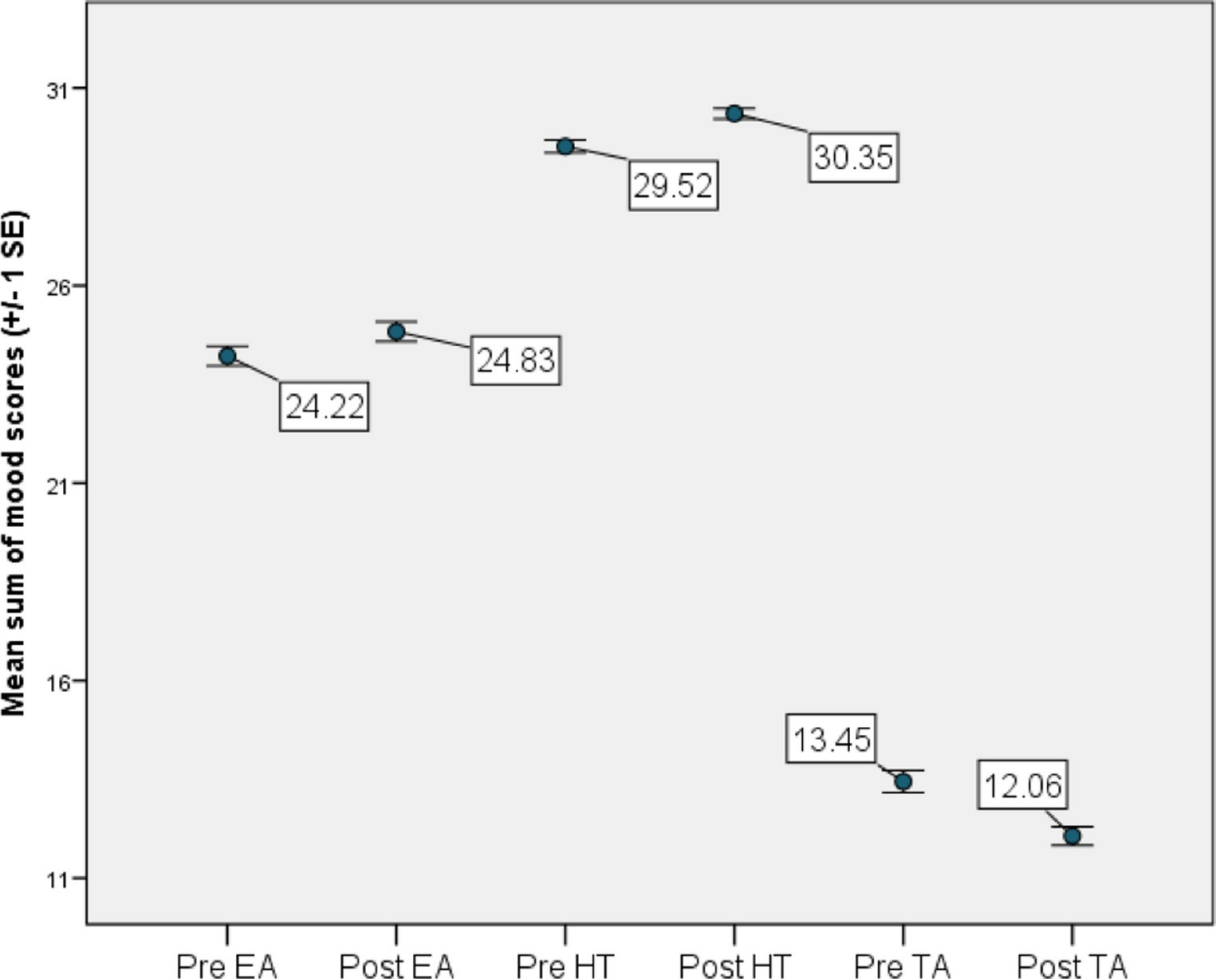
Study participant average UMACL scores for tense arousal (TA), energetic arousal (EA), and hedonic tone (HT) before and after the River’s Edge experience at the Saint Louis Zoo.

**Fig 7:**
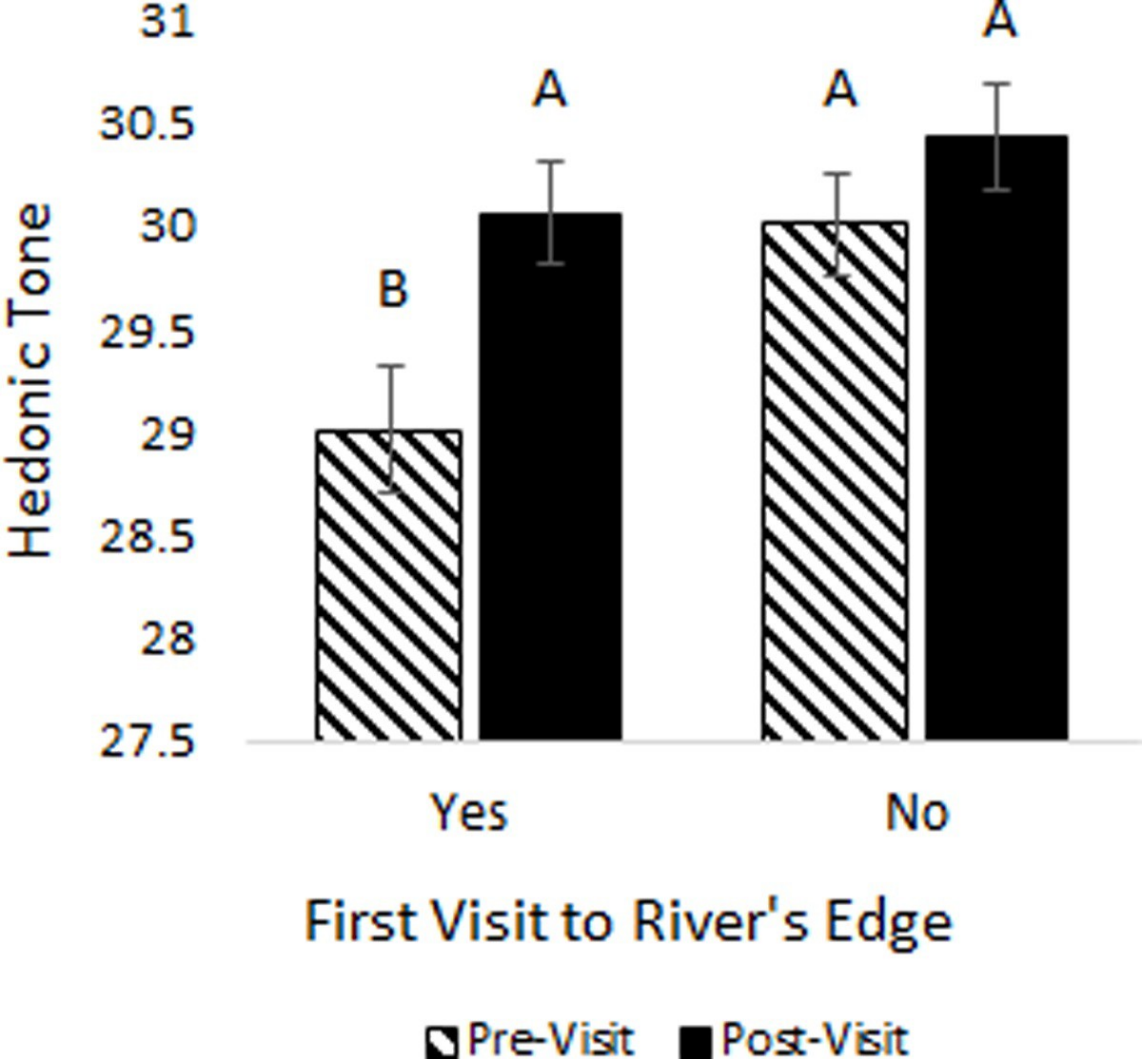
Study participants’ hedonic tone (HT) over time by first or return visit to the River’s Edge experience. Different letters designate significant differences based on the Tukey adjustment for multiple mean comparisons at α = 0.05.

On average, HT significantly increased from pre- to post-River’s Edge experience (*t*_185_ = -5.35, *p* = <0.0001, Fig 6). This relationship was still significant when the data were analyzed as a generalized mixed model (F_1,179_ = 9.56, *p* = 0.0023). Overall, HT was higher in people who had visited River’s Edge at least once before in the past (F_1,179_ = 6.09, *p* = 0.015), and there was a trend in which HT tended to increased more over time only in individuals who were visiting River’s Edge for the first time (F_1,179_ = 3.63, *p* = 0.059; Fig 7).

### Predictors of energetic arousal

On average, EA significantly increased from pre- to post-River’s Edge experience (*t*_185_ = -3.04, *p* = 0.0027, Fig 6). This relationship was only marginally significant when the data were analyzed as a generalized mixed model (F_1,179_ = 2.78, *p* = 0.0973). Similar to HT, EA was higher in people who had visited the River’s Edge at least once before (F_1,179_ = 8.07, *p* = 0.005) which was the only significant predictor of EA.

### Predictors of tense arousal

On average, TA significantly decreased from pre- to post-River’s Edge experience (*t*_185_ = 6.38, *p* = <0.0001, Fig 6). This relationship was still significant when the data were analyzed as a generalized mixed model (r = 0.0002, t_179_ = 2.46, *p* = 0.011). The magnitude of the decrease in TA from the time the participants entered River’s Edge to the time they exited River’s Edge was dependent on gender. Women had a greater decline in TA over time (pre to post) compared to men (F_1,179_ = 4.21, *p* = 0.042). TA decreased in women over time but did not change significantly over time in men. Other observed effects that were independent of time included a significant positive association between the daily Zoo attendance and the level of TA in participants; the higher the attendance the higher the TA (r = -0.2813, t_179_ = -2.45, *p* = 0.015). Similarly, people with a more negative parking rating also had higher TA (F_1,179_ = 6.0, *p* = 0.015). The number of exhibits seen prior to arriving at River’s Edge was negatively associated with TA (r = -0.2211, t_179_ = -2.11, *p* = 0.036), therefore, the more exhibits visited, the lower the TA.

**Fig 8:**
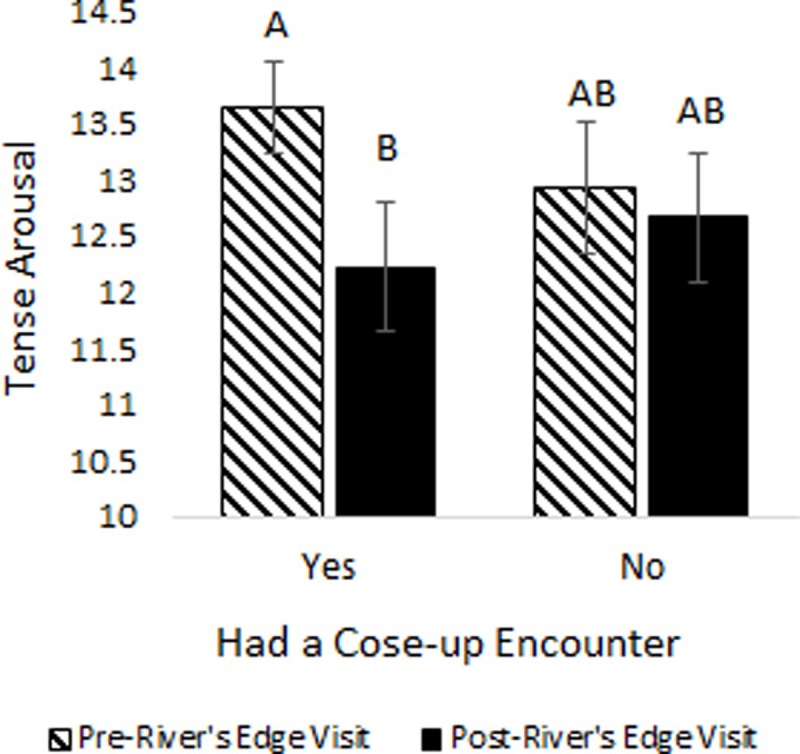
Study participants’ tense arousal (TA) over time by whether or not they had a close-up encounter with the most engaging animal during the River’s Edge experience. Different letters designate significant differences based on the Tukey adjustment for multiple mean comparisons at α = 0.05.

We found a positive association with TA for people that looked into the eyes of an animal they rated as having the most engaging experience with (F_1,179_ = 6.02, *p* = 0.015). People with higher TA more often reported looking into an animal’s eyes, but the experience itself did not affect the degree of change in TA between the pre to post experience. In contrast, people who reported having a close-up encounter with an animal they had “the most engaging experience with” during the River’s Edge experience had a significant decrease in TA over time. People without a close-up experience did not have a change in TA (t_179_ = -4.21, *p* = 0.0002 and t_179_ = -0.54, *p* = 0.95, respectively, Fig 8).

## Discussion

The goal of this study was to determine whether visitors walking through an immersive, naturalistic exhibit at the Saint Louis Zoo would experience beneficial physiological and/or psychological changes, and what might contribute to these changes. The results of the study support our hypotheses that visitors would have decreased salivary cortisol levels and blood pressure levels, while also reporting an increase in hedonic tone and energetic arousal and a decrease in tense arousal. This demonstrates a reduction in physiological and psychological stress for study participants.

Our findings support that visiting zoos benefit visitor health. Certain aspects of the zoo experience appear to drive these benefits. The more exhibits that participants reported seeing prior to attending River’s Edge, as well as individuals who had visited River’s Edge prior to this study, had a greater decrease in physiological and/or psychological stress indicators. Individuals that saw more exhibits had additional time to experience naturalistic habitats and green spaces at the Saint Louis Zoo and may have benefited from their stress-reducing effects prior to entering River’s Edge. Additionally, participants who had been to River’s Edge on a previous Zoo visit may have decided to return to River’s Edge because they had knowingly, or unknowingly experienced the stress-reducing effects that occurred during their prior visit.

Our findings support the role that zoos and other green spaces can play in benefitting human health through stress reduction, while also supporting the growing body of literature regarding the role of zoos in a One Health framework [27, 28]. As one of the few free zoos in the United States, the Saint Louis Zoo may also provide a unique research opportunity for exploring human health outcomes across a spectrum of socio-economic levels. Health disparities are evident in St. Louis; residents of neighboring zip codes only miles apart have been shown to have an 18-year difference in life expectancy, and it has been argued that these discrepancies must be addressed at all levels [53]. A recent national study found that only 54% of children from low-income households have visited a zoo or aquarium compared to 69% of children from high-income households [54]. Zoos, especially those with free admission, could increase access to improve health outcomes for visitors across socio-economic levels.

Using a One Health approach to demonstrate the benefits of zoos and other green spaces could be effective in these efforts [18].

There are a few limitations with this study. Each participant was used as their own control rather than having a separate control group that did not walk through an immersive, naturalistic zoo exhibit. Thus, we cannot claim that having this kind of experience provides a unique health benefit without comparison to individuals who might, for example, walk a nature trail in a park and/or a non-natural environment such as a shopping mall. Studies have shown that physical activity in natural environment have been associated with a greater reduction in poor mental health outcomes and improvements in self-reported well-being [55, 56]. Studies have found that older adults who engaged in mall walking, as an example of non-natural environment, reported stress reduction and uplifting moods as well as improvements in physical, social and emotional well-being [57, 58].

People who reported having a close-up encounter with the animal they found to be “most engaging” during their River’s Edge experience exhibited a significant decrease in TA. People without a close-up encounter with an animal did not have a change in TA, which suggests zoos or other places where people encounter animals up close are particularly beneficial for health. While close-up opportunities for viewing animals in zoos may provide human health benefits, animal welfare is the utmost priority at the Saint Louis Zoo. In accredited zoos and aquariums, animal habitats are designed to maximize animal welfare while providing an outstanding visitor experience. Animals are always given control over their proximity to humans.

A second limitation may have been our lack of assessment of health characteristics of the participants, including information about overall health and fitness. Health issues of participants may have impacted the results of this study. However we did ask participants pertinent questions related to health issues that had known impacts in skewing the results of our study including being on blood thinners (two participants) and/or undergoing treatment for high-blood pressure (six participants).

Despite the statistical significance, the biological significance of the physiological and psychological changes observed in this study are unclear. For example, the changes in diastolic and systolic blood pressure measures were approximately two mmHg. While two mmHg appears to be a small decrease, a sizeable percentage of participants shifted from a higher systolic and diastolic blood pressure category to the normal systolic and diastolic blood pressure category. A larger sample size may have revealed a more significant effect. Although these categorical changes are remarkable and suggest biological significance, it is not clear whether this is the case.

## Conclusions

Dan Ashe, President and CEO of AZA, argues “AZA-accredited zoos and aquariums are places where people of all ages, gender, races, religions, ethnicities, abilities and cultures create bonds with animals they will likely never have opportunity to see in the wild. In a world where people are increasingly urbanizing and disconnected from nature, aquarium and zoo experiences are providing connections to the natural world…” [59]. In addition to their roles in providing recreational opportunities, educating the public and inspiring them to take conservation action, recent research suggests zoos and aquariums can also offer tangible health benefits to visitors [25]. To enhance these benefits, zoos and other nature-based attractions can improve aspects of their institutions that are shown to increase stress (e.g., limited parking) and provide exhibit experiences that decrease stress (e.g., close-up viewing of animals). Studies such as ours provide evidence that zoos provide physiological and psychological human health benefits to visitors from all backgrounds. This reinforces the role of zoos within the growing One Health movement as zoos often blend environmental, human and animal health [18, 19, 20, 21].

We recommend that additional studies be conducted to look at the overall effects of zoos on human health. These will allow us to determine the role zoos may play in regards to human health, and specifically how zoos may better serve the physical and psychological health needs of people from diverse backgrounds, including marginalized populations who already experience significant health disparities.
